# Supplementary material for: Predicting visual function by interpreting a neuronal wiring diagram
Source: Nature. 2024 Oct 2;634(8032):113–23. doi: 10.1038/s41586-024-07953-5 (PMC11446822; doi:10.1038/s41586-024-07953-5)
Supplement: Supplementary file 6 — CRF and ERF predictions for individual TmY4 and TmY9 cells. Analogous to Supplementary Data 3, but for TmY target types. Shown are the top four monosynaptic pathways, the strongest pathway passing through each of the top ten intermediary types (ranking from Extended Data Fig. 7), and the trisynaptic pathway Tm1–TmY–Dm3–TmY (see the section entitled Prediction of spatial normalization). [file 41586_2024_7953_MOESM6_ESM.zip › DataS4/TmY9qΓèÑ/720575940627907752.pdf]

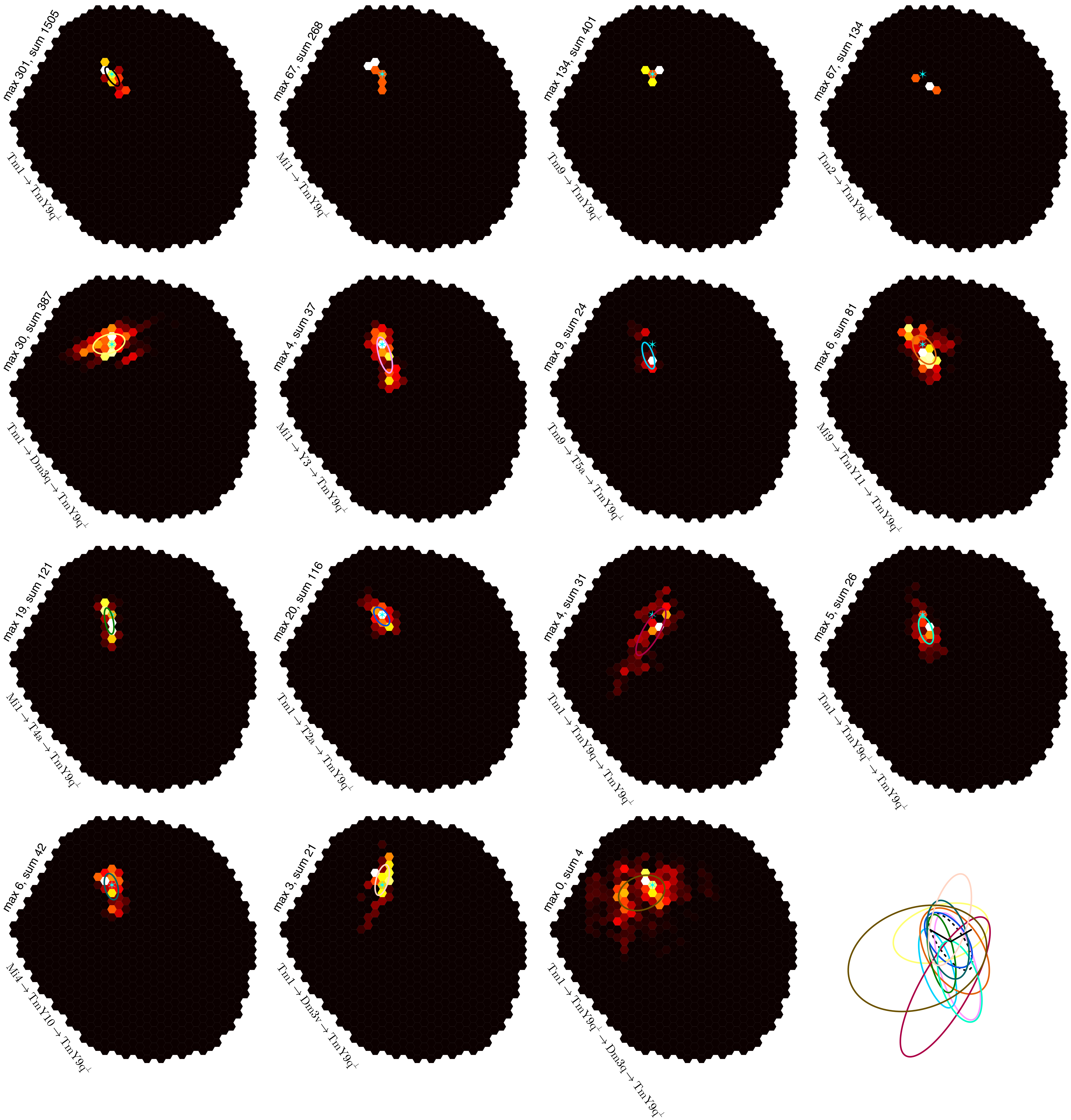
max 301, sum 1505
Tm1 → TmY9q⊥
max 67, sum 268
Mi1 → TmY9q⊥
max 134, sum 401
Tm9 → TmY9q⊥
max 67, sum 134
Tm2 → TmY9q⊥
max 30, sum 387
Tm1 → Dm3q → TmY9q⊥
max 4, sum 37
Mi1 → Y3 → TmY9q⊥
max 9, sum 24
Tm9 → T5a → TmY9q⊥
max 6, sum 81
Mi9 → TmY11 → TmY9q⊥
max 19, sum 121
Mi1 → T4a → TmY9q⊥
max 20, sum 116
Tm1 → T2a → TmY9q⊥
max 4, sum 31
Tm1 → TmY9q → TmY9q⊥
max 5, sum 26
Tm1 → TmY9q⊥ → TmY9q⊥
max 6, sum 42
Mi4 → TmY10 → TmY9q⊥
max 3, sum 21
Tm1 → Dm3v → TmY9q⊥
max 0, sum 4
Tm1 → TmY9q⊥ → Dm3q → TmY9q⊥
